# Rice Husk Ash to Stabilize Heavy Metals Contained in Municipal Solid Waste Incineration Fly Ash: First Results by Applying New Pre-treatment Technology

**DOI:** 10.3390/ma8105346

**Published:** 2015-10-09

**Authors:** Laura Benassi, Federica Franchi, Daniele Catina, Flavio Cioffi, Nicola Rodella, Laura Borgese, Michela Pasquali, Laura E. Depero, Elza Bontempi

**Affiliations:** 1 INSTM and Chemistry for Technologies Laboratory, University of Brescia, via Branze 38, Brescia 25123, Italy; l.benassi001@unibs.it (L.B.); f.franchi005@studenti.unibs.it (F.F.); d.catina@studenti.unibs.it (D.C.); n.rodella@unibs.it (N.R.); laura.borgese@unibs.it (L.B.); michela.pasquali@unibs.it (M.P.); laura.depero@unibs.it (L.E.D.); 2 Contento Trade srl, Via Vieris 11, Terenzano (UD) 33050, Italy; flavio@contentotrade.net

**Keywords:** fly ash, rice husk ash, DIC, heavy metals stabilization, COSMOS-RICE

## Abstract

A new technology was recently developed for municipal solid waste incineration (MSWI) fly ash stabilization, based on the employment of all waste and byproduct materials. In particular, the proposed method is based on the use of amorphous silica contained in rice husk ash (RHA), an agricultural byproduct material (COSMOS-RICE project). The obtained final inert can be applied in several applications to produce “green composites”. In this work, for the first time, a process for pre-treatment of rice husk, before its use in the stabilization of heavy metals, based on the employment of Instant Pressure Drop technology (DIC) was tested. The aim of this work is to verify the influence of the pre-treatment on the efficiency on heavy metals stabilization in the COSMOS-RICE technology. DIC technique is based on a thermomechanical effect induced by an abrupt transition from high steam pressure to a vacuum, to produce changes in the material. Two different DIC pre-treatments were selected and thermal annealing at different temperatures were performed on rice husk. The resulting RHAs were employed to obtain COSMOS-RICE samples, and the stabilization procedure was tested on the MSWI fly ash. In the frame of this work, some thermal treatments were also realized in O_2_-limiting conditions, to test the effect of charcoal obtained from RHA on the stabilization procedure. The results of this work show that the application of DIC technology into existing treatment cycles of some waste materials should be investigated in more details to offer the possibility to stabilize and reuse waste.

## 1. Introduction

Because of poor combustion efficiency and the lack of air pollution control systems, from its early beginning in the 1870s, waste incineration was considered a very large source of pollution [1]. From 1950, the first efforts to limit the pollution of incinerators were imposed by law [2]. However these first limitations did not consider dust emissions, until 1970, when an aggravation of dust emission standards occurred. As a consequence, in the next years, the development of the air pollution control systems started [1].

Today, while modern waste incineration represents a consolidate technology for volume and mass reduction of the waste, combined with efficient energy recovery and systems to reduce and treat emissions, some concerns regarding the production of toxic waste still exist. In particular, fly ash generated by the air pollution control systems are hazardous waste materials for which landfilling is still considered the most appropriate management strategy. Indeed, this ash contains leachable toxic metals, as for example Pb and Zn [3].

Great efforts of researchers are now devoted to developing sustainable stabilization technologies able to reuse this toxic ash, also in view of the European need of new solutions for raw materials substitution that are employed in several applications.

In recent works, we proposed the use of silica extracted from rice husk ash (RHA) for heavy metals stabilization of municipal solid waste incineration (MSWI) fly ash [4]. The new proposed technology was named COSMOS-RICE.

The idea to use this agricultural byproduct is due to its high availability. World rice production in 2011–2012 exceeded 480 million tons [5]. Rice husk constitutes about 20 wt % of the paddy production and it is an abundantly available biomass, commonly employed as a fuel. Its combustion product, rice husk ash (RHA), usually contains more than 60% silica (SiO_2_), and 10%–40% carbon and other minor mineral components. Due to the lower density of RHA, and consequently its bulky form, its disposal can become a problem. In some areas, a large amount of RHA is treated as a waste and landfilled, leading to air and water pollution. Airborne RHA particles have been linked to respiratory disease in humans [6]. Because rice husk is abundantly available and it can be converted into RHA after being subjected to an almost zero-cost thermal valorization process, more and more researchers have become interested in how to use this waste as a resource [7].

The stabilization mechanism involved in the COSMOS-RICE technology was extensively discussed [8]: it is attributed to two different reactions, due to the amorphous silica and carbonation. Indeed, the stabilization mechanism is mainly due to the capability of amorphous silica to entrap heavy metals, but this does not only occur during laboratory treatment, it also proceeds over time thanks to carbonation. The proposed method was very recently improved by the direct use of RHA without passing through silica extraction [8]. Since the organic part of rice husk (RH) can easily be removed by thermal decomposition, thermal treatment of RH is an approach to obtain, not only energy, but also silica from rice husk.

Production of biochar from rice husk has also attracted many researchers’ attention. This is a versatile material with good adsorption properties. Biochar has a relatively structured carbon matrix with a high degree of porosity and an extensive surface area. It may act as an adsorbent, which is similar in some aspects to activated carbon and it can play an important role in controlling contaminants in the environment [9]. Chemical impregnation with KOH or NaOH of RHA, followed by activation at 650–850 °C, generates a material with extremely high surface area [10]. Even though biochar from rice husk has already been employed for removing heavy metals [11], it was never considered in the COSMOS-RICE technology.

To also obtain biochar from RHA, pyrolysis of rice husk was realized at 500 °C in O_2_-limiting conditions.

To evaluate the effect on heavy metals stabilization by a pre-treatment of rice husk, the employment of Instant Pressure Drop (DIC) technology [12] was proposed for the first time.

The DIC technology is based on a thermo-mechanical process that requires some moisture level in the material to be treated. A rapid pressure drop occurs during the material heating, producing bursts of moisture evaporation inside the bulk of material, with the occurrence of a structural damage. It was reported that DIC pre-treatment is able to make a material porous and expanded within short treatment time [13]. This process is based on the use of vacuum and temperature. Moreover, material properties are fundamental for obtaining different final characteristics of the product.

Several research papers have been published in recent years exploring the use of DIC technology, in particular for food treatment [14]. Some applications that have been investigated include material sterilization, removal of certain inhibitors in legumes, structural expansion of fruit and vegetables, drying process acceleration, enhancement of extraction of essential oils, coffee extraction, and preservation of food materials [15,16,17]. DIC technology was also applied on rice preservation [18]. However, it was never employed as a pre-treatment of rice husk.

This paper reports the first work about the application of DIC as a possible new pre-treatment technology for the waste material. The aim of this project is to obtain a safe filler that can be applied in several application, to produce “green composites”. This pioneering work explores the viability of applying DIC technology to pre-treat other waste types.

## 2. Materials and Method

### 2.1. Pretreatment of Rice Husk Samples by DIC Technology

Rice husk was provided by an Italian factory (Lombardy region).

DIC technology is extensively described in several papers (see the Introduction Section). In the present work, we employed an experimental reactor designed and realized by Contento Trade srl.

The laboratory DIC plant is composed of a 5 L stainless steel reactor, a refrigerated stainless steel vacuum chamber with 500 L of capacity, a vacuum pump, a steam generator and a steam overheater, and is completely controlled by software developed with Labview. It is able to carry out simple or cyclic DIC tests up to a maximum temperature and pressure of 180 °C and 16 bar, respectively.

**Figure 1:**
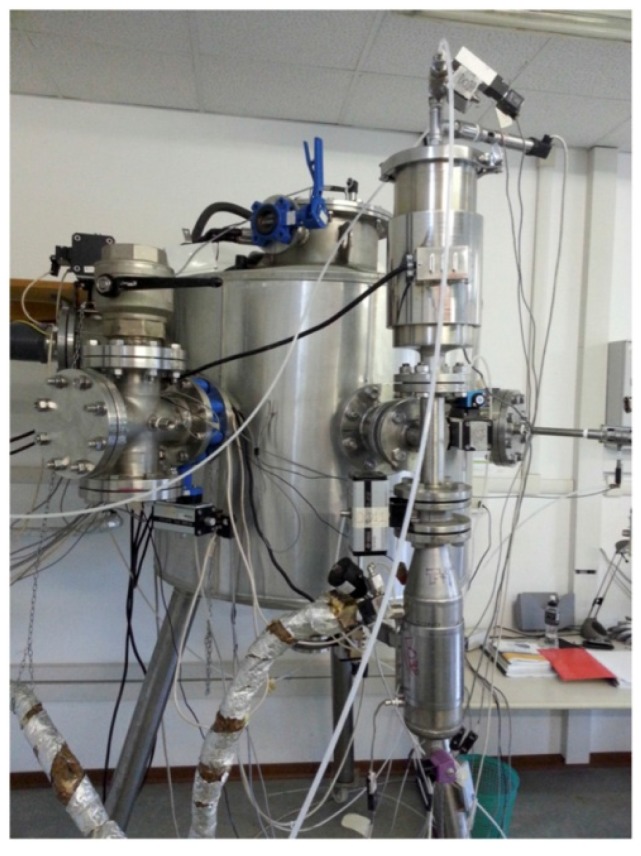
Picture of the employed experimental DIC instrument.

Figure 1 shows a picture of the instrument.

For the DIC pre-treatment, rice husk samples were enclosed in the stainless steel container at atmospheric pressure and then this was closed. An initial vacuum was performed. Afterwards, saturated steam was injected into the reactor. The abrupt pressure drop towards a vacuum was carried out by an abrupt opening (<0.2 s) of a valve connecting the reactor with the steel vacuum chamber. This abrupt adiabatic pressure drop triggered auto-vaporization of superheated water contained in the material, instantaneous cooling, structure swelling, and even rupture of the structure.

The best texturing process allows obtaining the final product at lower density and with less residual water content. For this aim, several DIC pre-treatments were tested, changing pressure steam, temperature in the chambers, maximum applied pressure, conditioning time and number of repetitions. Based on these preliminary tests, two DIC pre-treatments were identified as the most suitable to obtain a final moisture content of about 10%. After the DIC pre-treatments, samples were thermally annealed in a Nabertherm electric oven at different temperatures for 270 min (see Table 1 at the end of the experimental section).

Pyrolysis of rice husk, to obtain a carbon-rich solid residue, was realized with thermal treatments at 500 °C in sealed sample-holders (*i.e.*, under O_2_-limited conditions), inserted in the oven.

Table 1 summarizes the samples description and treatments. Samples that have been pre-treated by DIC were named T1 and T2. Samples that were not pre-treated are named TQ.

**Table 1:** Samples description and treatments. All the thermal annealing were made for 270 min.

Sample ID	DIC Treatment	Annealing Temperature (°C)	O_2_-Limited Conditions	
TQ 500	NO	500	No	
TQ 550	NO	550	No	
TQ 600	NO	600	No	
TQ 500 B	NO	500	Yes	
T1 500	T1	500	No	
T1 550	T1	550	No	
T1 600	T1	600	No	
T1 500 B	T1	500	Yes	
T2 500	T2	500	No	
T2 550	T2	550	No	
T2 600	T2	600	No	
T2 500 B	T2	500	Yes	
**DIC Treatment**	**Steam Pressure**	**Max. Temperature**	**Max. Pressure**	**Cycle Repetitions**
T1	3 bar	150 °C	0.075 br	1
T2	3 bar	150 °C	0.050 bar	3

### 2.2. Determination of Si in Rice Husk Ash Samples

To evaluate the silica content in RHA, a chemical extraction procedure was realized. Fifty grams of 1 N NaOH were added to 0.5 g of RHA in a low density polyethylene container maintained at room temperature for 24 h under constant agitation to dissolve the silica and produce a sodium silicate solution; then, the solutions were filtered and analyzed by ICP-MS to determinate the silica content according to EPA 6010C 2007 method. The extracted silica content resulted 47% (±12%) and 23% (±2%) for samples treated in the open and sealed sample-holders, respectively. These results highlight the lower content of the extracted silica in samples thermally treated in oxygen deficient conditions, in respect to the samples thermally treated in air.

### 2.3. Morphological and Structural Characterization of Samples

Morphologic characteristics of rice husk ash sample were investigated by SEM and XRD. X-Ray Diffraction (XRD) measurements were performed with Panalytical X’Pert Pro diffractometer (University of Brescia, Brescia, Italy) equipped with the X’Celerator detector and Cu anode. Operating values were 40 kV and 40 mA.

The rice husk ashes morphology was investigated with Scanning Electron Microscopy, LEO EVO 40 (University of Brescia, Brescia, Italy) equipped with an energy dispersive microprobe (EDS) quantitative analysis.

### 2.4. Stabilization of Municipal Solid Waste Incineration Fly Ash

Stabilized samples made from MSWI fly ash by means of COSMOS-RICE technology were prepared by adding to the MSWI fly ash (provided by an Italian incinerator), other waste materials: *i.e.*, coal fly ash, rice husk ash (RHA), and flue gas desulfurization (FGD) residues, in accordance to the already established procedure [8]. All the samples were prepared following the same protocol, *i.e.*, by adding to a mixture of the three powders, MSWI fly ash, FGD residues, and coal fly ash (in the weight quantity of 130 g, 10 g, and 15 g, respectively), 1.6 g of RHA (about 1% of the total weight of ash), wetting with 170 g of water, and stirring for 30 min in a laboratory mixer at 60 °C [4]. Generally, the quantity of added silica in the COSMOS technology, ranges between 5% and 10% of the total weight of ash. The lower quantity of RHA used in this work was chosen to be sure that the complete stabilization of heavy metals does not occur. This is necessary to compare the results obtained by using different RHA pre-treatment conditions and to select the best pre-treatment.

After chemical stabilization of heavy metals, the solid inertized material can be washed out to recover almost pure soluble salts [19]. In the present case, salts recovery was not realized.

### 2.5. Leaching Tests

Leaching tests were performed when samples resulted completely dried (30 days after the stabilization treatment).

Leaching tests were carried out according to the CEN normative [20], in order to quantify the leachability of heavy metals in water. Tests were performed mixing the dried and homogenized sample with MilliQ water at a liquid to solid ratio of 10 L/kg. The particle size was below 4 mm (with size reduction) and a temperature in compliance with the Directive (about 20 °C). The contact time of materials and aqueous solution was 2 h. This time was considered sufficient to establish equilibrium in subsequent experiments with MSWI fly ash [21].

Chemical analysis of the leachate solution was done by means of Total Reflection X-Ray Fluorescence technique (TXRF) by the Bruker TXRF system S2 Picofox (University of Brescia, Brescia, Italy) (air cooled, Mo tube, Silicon-Drift Detector), with operating values of 50 kV and 750 μA using an acquisition time of 600 s. TXRF quantitative analysis of the suspended samples was performed by the internal standard procedure. A proper amount of gallium, used as an internal standard element, was added [22]. Every analysis has been replicated three times.

## 3. Results and Discussion

**Figure 2:**
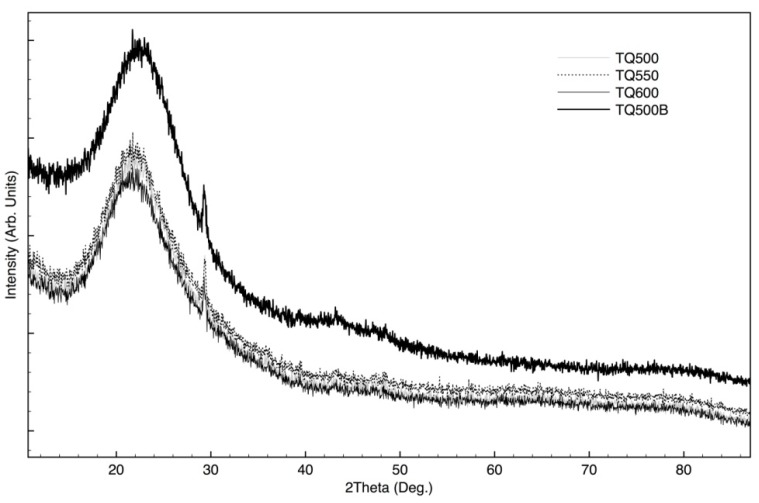
X-ray diffraction analysis of TQ rice husk ashes treated at different temperatures (see Table 1).

Figure 2 shows X-ray diffraction analysis of TQ rice husk ash treated at different temperatures and oven conditions (see Table 1). The XRD patterns of samples T1 and T2 (DIC pre-treated samples), thermally treated in the same conditions are very similar to corresponding reported TQ samples, hence, for the purposes of clarity, they were not shown in the Figure. From XRD patterns shown in Figure 2, it appears that all samples are amorphous, with one peak, due to the presence of a crystalline phase.

Despite the fact that all patterns can essentially be attributed to the presence of amorphous materials, it is evident that basic materials structures are different: indeed, the integrated areas of the XRD patterns are almost the same for samples treated in O2-limited conditions, but they are extremely different from those regarding samples not treated in O2-limited conditions. Moreover, for the last samples, the integrated XRD area is almost the same.

Samples TQ 500, 550 and 600 show a large and intense halo, in the range of 18°–28°. In the literature it was reported that, for similar samples, the typical silica oxide characteristic broad peak is centered at 2θ = 22.5°, which can be attributed to the presence of disordered cristobalite [23]. Moreover, the sample TQ 500 B shows a different XRD spectra, with higher intensities, compared to the other shown in the same Figure (the XRD spectra of T1 600 B and T2 600 B are very similar, then are not reported).

The background intensity of sample TQ 500 B, which is extremely high in respect to other samples, can be caused by the amorphous carbon present in the RHA [24].

In particular, for sample TQ 500 B there is a large halo between 16° and 30°), which can be attributed to (002) stacking of the graphitic basal plans in chars [25,26]. Indeed, this halo results at slight higher diffraction angles, in comparison to peak attributed to the silica.

Regarding sample TQ 500 B, another large halo can be found between 38° and 50°. For carbon prepared at different pyrolysis temperatures and for commercial carbon black, X-ray diffraction patterns show two peaks at about 24° and 44°, which were assigned to the planes of graphite (002) and (100) [27].

Therefore, XRD patterns collected on RHA allow concluding that samples annealed in not O2-limited conditions are mainly made of amorphous silica. On the contrary, samples obtained in O2-limited conditions show an important content of biochar. These results are in accord with data about silica content, obtained by NaOH solubilization. Indeed, the content of silica resulted extremely lower for samples treated in the O2-limited conditions, in respect to other samples (see the Experimental Section).

RHA powders were employed for the COSMOS-RICE stabilization tests conducted with MSWI fly ash.

Table 2 reports the elements concentration in the solutions obtained by making the leaching test on MSWI fly ash, before the stabilization treatment. It appears evident that solution contains some metals. In particular, the toxic elements that can be found in high concentration are Pb and Zn. As it be seen in Table 2, the concentrations of Pb and Zn are 60 and 14 mg/L, respectively.

**Table 2:** Elements concentration and limits of detection (LD) in leaching solutions of MSWI fly ash.

Element	Conc. (mg/L)	LD (mg/L)
Cl	9287 ± 928	0.161
K	1385 ± 138	0.092
Ca	6870 ± 687	0.068
Zn	14.3 ± 1.4	0.004
As	1.6 ± 0.2	0.004
Br	118 ± 12	0.004
Rb	4.0 ± 0.4	0.003
Sr	6.2 ± 0.6	0.003
Ba	8.7 ± 0.9	0.033
Pb	60 ± 6	0.004

**Figure 3:**
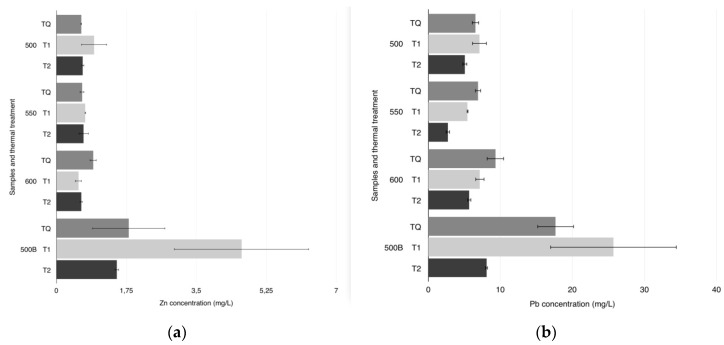
Concentration of Zn (**a**) and Pb (**b**) in the leaching solutions of the COSMOS-RICE samples obtained by using the whole RHA samples (see Table 1).

To verify the stabilization efficacy of the different obtained RHAs, one month after the stabilization procedure (samples were left to dry at room temperature for one month) leaching tests were realized. The data about the concentration of Pb and Zn in the leaching solutions of stabilized samples are shown in Figure 3.

It is very important to highlight that the COSMOS-RICE conditions of synthesis were settled to obtain incomplete stabilization reactions: *i.e.*, the amount of added RHA was about 1% of the total weight of ash and thus, was much lower than that employed in previous works (around 20% in weight of the global fly ash mix) [4,19,28]. This allows finding residual soluble metals in the leaching solutions, to have the possibility to compare the results obtained by using RHA treated in different manners and discriminate the efficacy of different employed RHAs. As a consequence, the samples that have been most effectively stabilized exhibit lower Pb and Zn concentrations in their leaching solutions (see Figure 3). Comparing all the data shown in Figure 3, it results that, despite the low amount of used RHA, the stabilization of MSWI fly ash was obtained: in the leaching solutions, Zn and Pb were reduced at values generally less than one order of magnitude (for example, in sample T2 550, Pb was about 3 mg/L and Zn 1 mg/L). 

In particular, there are two effects to investigate: the presence of biochar in the RHA and the DIC pre-treatments. The first consideration that can be deduced by comparing the data reported in Figure 3 concerns the biochar: RHAs that also contains biochar (see the corresponding stabilized samples, TQ 500 B, T1 500 B, and T2 500 B) are globally less efficient in the heavy metals stabilization because of the higher amount of Pb and Zn found in the leaching solutions. On the basis of these results, it is possible to conclude that the stabilization efficacy of COSMOS-RICE technology is reduced when silica content in RHA is decreased. This is an unexpected result, because there are several examples of use of biochar derived from rice husk as metal absorber reported in the literature [9]. However, the data shown in Figure 3 highlight that the stabilization is better obtained by silica in respect to biochar contained in RHA.

The data in Figure 3 are analyzed in more details below, in order to evaluate the role of the DIC treatment in the stabilization process.

Considering Zn, its concentration is very low (it is generally lower than 1 mg/L), except for samples TQ 500 B, T1 500 B, and T2 500 B, where the RHA do not contain mainly silica but also biochar.

Because of the Zn concentration values are very low, it is not possible to enhance a role of DIC pre-treatment in the stabilization of Zn. However, in the case of samples also containing biochar, it seems that T2 DIC pre-treatment is more efficient in comparison to T1 and not DIC not pre-treated sample (TQ).

Data about Pb concentration in the leaching solutions of stabilized samples are about one order of magnitude higher than Zn. Then, some differences about results of stabilization performed using differently treated RHA are more evident. Considering RHA samples treated in the same thermal conditions, but with different pre-treatment, it appears that leaching solution of samples pre-treated with DIC (T1 and T2) show lower Pb concentration, in respect to TQ (except for samples TQ 500 B, T1 500 B, and T2 500 B). In addition, it clearly appears that T2 pre-treatment results the most efficient in all cases. This is probably also due to the fact that T2 involves three DIC cycle repetitions, and thus it resulted more incisive.

The slight differences among the effectiveness of stabilization procedure for all T1 (and T2) samples can be due to the thermal annealing temperature. Generally, a higher annealing temperature allows obtaining a higher silica content in the RHA, which allows a better heavy metals stabilization [29], in respect to lower annealing temperatures. Indeed, the degradation of organic part present in rice husk occurs starting from 350 °C until about 480 °C [30]. On the other hand, an increase of the annealing temperature can promote the amorphous silica crystallization, with a reduction of the amount of reactive silica. As a consequence, it is extremely difficult, at these temperatures, to have an idea of the predominant factor, related to the amount of amorphous silica present in the RHA.

**Figure 4:**
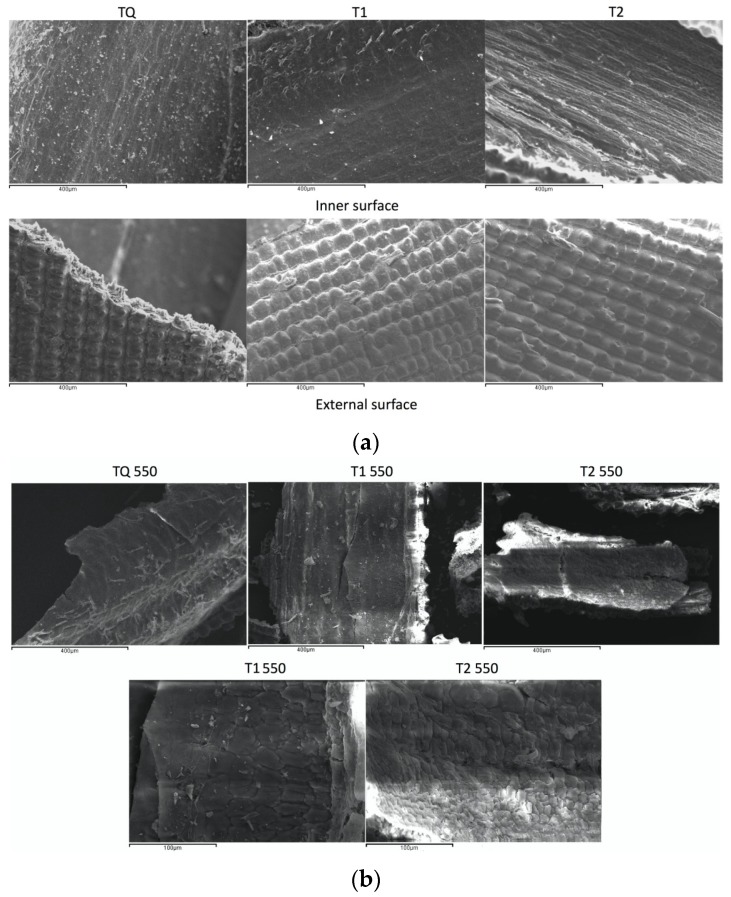
(**a**) SEM images of internal and external surfaces of untreated rice husk (TQ) and pre-treated by T1 and T2 DIC treatments. (**b**) SEM images of internal surfaces of corresponding RHA shown in Figure 4a.

SEM images of pre-treated rice husk samples and thermally annealed in different conditions are reported in Figure 4.

Figure 4a shows the inner and external surfaces of rice husk samples, not pre-treated and pre-treated DIC. As revealed in the SEM image, the external surface of the whole rice husk samples is covered with small irregularly shaped particles that are well aligned. Such domes are shown to be rich in silica. On the contrary, the internal surface of rice husk looks much smoother, and contains a much lower concentration of silicon [31]. External surfaces of all samples do not show any apparent morphological difference. Instead, Figure 4a highlight that internal surface of sample T1, and especially sample T2, seems to be more structured in respect to that of TQ sample, with the appearance of some channels. Since the internal part of rice husk contains less amount of silica, it is possible to conclude that DIC seems to produce some structural changes on the organic part of the sample. Indeed, for organic matter it often produces a structure swelling.

Figure 4b reports the SEM images of corresponding samples of Figure 4a, but after thermal treatment. It was chosen to show the morphology of RHA samples TQ 550, T2 550 and T1 550, to highlight possible morphological differences between samples pre-treated by DIC and not pre-treated (TQ), on the basis of leaching tests results reported in Figure 3. External surface images are not reported because no differences can be highlighted in the SEM images. It appears evident that, comparing the TQ 550 samples with the corresponding two other samples pre-treated by DIC technology, some morphological differences occurred. Sample T1 550 shows a more structured morphology, very similar to that one obtained for sample T2, before making any thermal annealing (see Figure 4a). The appearance of some internal channels occurs that result more evidently in respect to T2 sample. This is probably due to thermal treatment at 550 °C, which produce degradation of organic part of rice husk. Indeed, this occurs starting from 350 °C till about to 480 °C [30], and that may happen in a different manner in respect to TQ sample, because the DIC pre-treatment is done.

Sample T1 and T2 550 have large internal cavities and a more open structure. In order to highlight this strange morphology for these samples, some images were also taken at high magnification (see Figure 4b). These images reveal a great impact of DIC on the microstructure of pre-treated samples. It can be noted that samples T1 and T2 550 show an inner morphology, made by irregular particles with slit-shaped surfaces that provide an internal porous structure producing a higher surface area in respect to TQ sample. In particular, the sample that shows best results in terms of heavy metals stabilization (T2 550) shows particles with dimensions lower in respect to T1 550. This may results in a better RHA degradation and then, in a higher reactivity of RHA.

**Figure 5:**
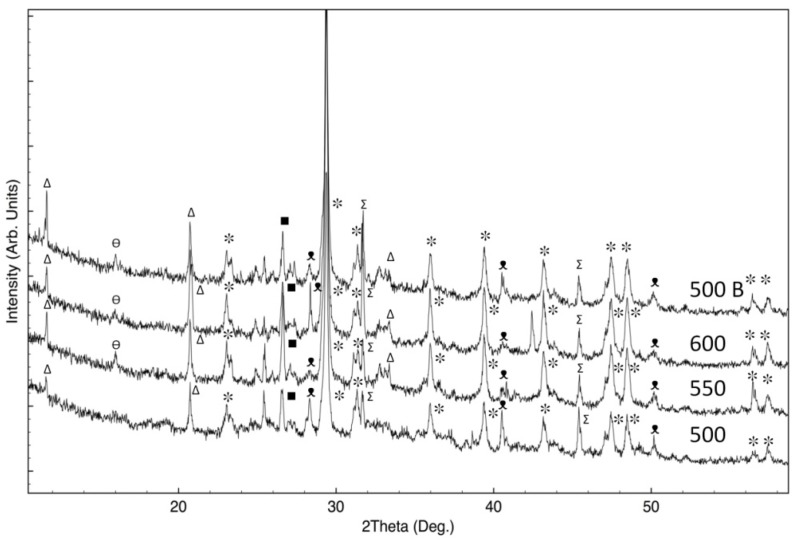
XRD patterns collected on COSMOS-RICE samples, obtained by using TQ RHA (see Table 1). ✻: Calcite (CaCO_3_); Σ: Halite (NaCl); Δ: Gypsum [CaSO_4_·2(H_2_O)]; θ: Thaumasite [Ca_3_(SO_4_)[Si(OH)_6_](CO_3_)·12(H_2_O)]; ▪: Quartz (SiO_2_); ᴥ Potassium chloride (KCl).

Figure 5 shows the XRD patterns collected on COSMOS-RICE samples, obtained by using TQ RHA. All COSMOS-RICE samples, obtained by employing the RHA reported in Table 1, show very similar XRD patterns. For this reason, only those of stabilized samples obtained by using TQ RHA are shown.

It is possible to see that all the samples contain calcite, gypsum, quartz, sylvite, halite, and thaumasite; like the phase mineralogy already reported for samples obtained by COSMOS-RICE technology. These phases are compatible with the employ of this material as a filler, to produce several composites. The stabilization mechanism involving amorphous silica reactions and carbonation has been already reported and discussed [8]. In particular, data shown in Figure 5 allows highlighting that the use of RHA obtained by different DIC pre-treatments does not produce any changes of the crystalline phases in the final stabilized materials. Indeed, DIC may influence the combustion efficiency of rice husk: it was shown that the RHA morphology appears more structured with consequences on the surface area. Therefore, at the same temperature, the combustion of organic part may result more efficient in samples pre-treated by DIC, with an increase of the silica concentration.

Work is in progress to evaluate the capability of DIC pre-treatment to increase the calorific efficiency of rice husk.

**Figure 6:**
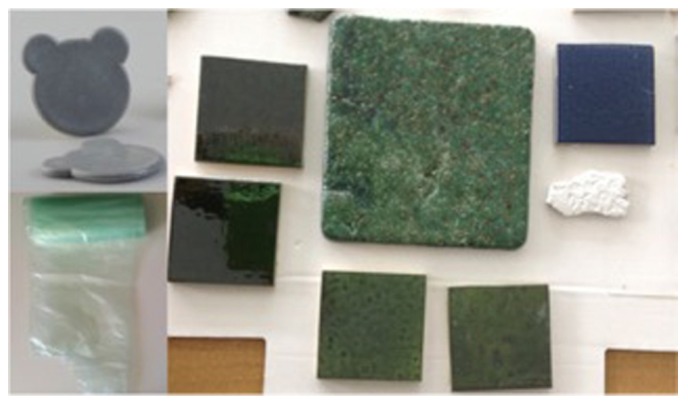
Pictures of several “green composites” realized by using the material produced with the COSMOS-RICE technology as a filler. It was inserted in polyethylene (30% in weight) and polyethylene foils (5% in weight). In addition, it was also employed to obtain tiles, with different colors (from 30% to 50% in weight).

Figure 6 show some “green composites” realized by using COSMOS-RICE technology, to obtain a new filler material. The new filler was inserted in polyethylene (30% in weight) and polyethylene foils (5% in weight). In addition, it was also employed to obtain tiles, with different colors (from 30% to 50% in weight). The presence of soluble salts may affect the employment of this material in building application. However, it was already shown that it is possible to wash the filler and recover almost pure soluble salts [19].

## 4. Conclusions

This work investigated the efficiency change of the COSMOS-RICE stabilization procedure, by increasing the biochar quantity contained in the RHA and also by a pre-treatment of rice husk, made by DIC technology. The aim is to produce a filler to be employed to produce new composites.

The increased amount of biochar in the RHA, in respect to the silica content, it is shown to have a negative impact in the stabilization of heavy metals.

On the contrary, the capability of DIC pre-treatment to increase the RHA stabilization of heavy metals was shown. This resulted more evident in the RHA samples containing biochar, which were characterized by high values of Zn and Pb in the leaching solutions.

On the basis of these results, it is possible to conclude that the DIC technology, developed to pre-treat food, can also be applied to pre-treat agricultural waste materials. In addition, the pre-treatment of other waste typologies should be investigated.
